# Supplementary material for: Uncertainties regarding the natural mortality of fish can increase due global climate change
Source: PeerJ. 2023 Mar 7;11:e14989. doi: 10.7717/peerj.14989 (PMC10000307; doi:10.7717/peerj.14989)
Supplement: Supplemental Information 1 [file peerj-11-14989-s001.docx]

# Supporting information

**S1 Appendix. Foundations of the fuzzy set theory.**

The difference between classical theory and fuzzy set theory begins in the concept of pertinence of an element to a set. In the classical logic of sets, the concept of pertinence of an element to a set is well defined. The degree of membership is expressed mathematically by the characteristic function, the definition of which is given below.

Definition (1) [Characteristic Function]: given a set *A* in a universe *U*, an element belongs to or does not belong to that set and cannot be partially contained:

$$\chi_{A}\left( x \right)=\left\{ \begin{aligned} 1 \mathrm{se} x\in A \\ 0 \mathrm{se} x\notin A \end{aligned} \right.$$

However, there are cases in which the degree of membership between elements and sets is not accurate, or rather, it is not known whether an element actually belongs to a set or not. Zadeh (1965) proposed a broader characterization, and generalized the characteristic function so that it could assume an infinite number of values in the interval [0.1].

Definition (2) [Fuzzy Set]: A fuzzy set *A* in a universe *U* is characterized by a membership function *φ*_A_: *U* → [0,1]. The membership value *φ*_A_(𝑥) indicates the degree of pertinence of *x* in *A*, where *φ*_A_(𝑥) = 0 and *φ*_A_(𝑥) = 1 indicate the non-pertinence and complete pertinence of *x* to the fuzzy set *A*, respectively. The fuzzy set *A* can be represented by a set of ordered pairs:

$$A= \{(x, \varphi A(x)), with x \in U\}$$

Definition (3) [Support of a Fuzzy Set]: Given a fuzzy set *A* in a universe *U*, the support of *A*, denoted by supp(*A*), is the (classical) subset of *U* defined by supp(*A*) = {*x* ∈ U : *φ*_A_(𝑥) > 0}. In general terms, supp(*A*) is the classical set of all elements that have a certain (non-zero) degree of pertinence in *A*.

Definition (4) [α-level]: Let *A* be a fuzzy subset of *U* defined by the relevance function *φ*_A_ : *U* → [0.1] and α ∈ [0.1]. The α-level of *A*, denoted by [*A*]*^α^*, is the classic subset of *U* defined by:

$$[A]^{\alpha}=\left\{ x\in U : \varphi_{A}\left( x \right)\geq\alpha\right\} for 0< \alpha\leq1$$

Definition (5) [Fuzzy Number]: A fuzzy subset *A* is called a fuzzy number when the universe set in which φ_A_ is defined is the set of the real numbers R and satisfy the following conditions:

(i) all the α-levels of *A* are non-empty, with 0 ≤ α ≤ 1;

(ii) all the α-levels of *A* are closed intervals of R;

(iii) supp*A* = {*x* ∈ R : φ_A_ (*x*) > 0} is limited.

Definition (6) [Zadeh’s Extension Principle]: Let *f* : *X* → *Z* and *A* be a fuzzy subset of *X*. Zadeh’s extension of *f* is the function $\hat{f}$ which, applied to *A*, provides the fuzzy subset $\hat{f}\left( A \right)$ of *Z*, whose membership function is defined as:

|  | $\varphi_{\hat{f}\left( A \right)}\left( z \right)=\left\{ \begin{matrix} \sup_{f^{-1}\left( z \right)} \varphi_{A}\left( x \right) if f^{-1}\left( z \right) \\ 0, if f^{-1}\left( z \right) \end{matrix} \right.$ |  |
| --- | --- | --- |

where 𝑓^−1^(*𝑧*) = {*x*; *f*(*x*) = *z*}, it is called the pre-image of z.

**S2 Appendix. Fuzzy Set for natural mortality of each species (pseudocode).**

**[** Parameter Definition **]**

**S** : array (mortality space).

var $\boldsymbol{L}_{\boldsymbol{\infty}}$ : Real (theoretical maximum length).

var $\boldsymbol{k}$ : Real (individual growth coeficiente).

$\boldsymbol{\Delta}_{\boldsymbol{i}}$ : array (interval for the i-th IPCC scenario, $i=1,2,3,4$).

${\tilde{\boldsymbol{\Delta}}}_{\boldsymbol{i}}$ : Fuzzy Set associated with the range $\Delta_{i}$.

$\tilde{\boldsymbol{T}}$ : Fuzzy Set associated with the average annual temperature.

${\tilde{\boldsymbol{T}}}_{\boldsymbol{i}}$ : Fuzzy Set for average annual temperature under the presence of the i-th scenario.

var **tmptr** : Real (the evaluated temperature).

var **valueM** : Real (numerical value for mortality).

$\boldsymbol{invF}\boldsymbol{p}$ : Object containing the inverse function obtained with Pauly's equation

**temperatureData** : Data Matrix (Database).

**scenarioGroups** : array ("grouping of IPCC scenarios").

**IC** : array ; Confidence interval array (Buckley, 2005).

**CC** : array ; Confidence coefficient associated with the confidence interval.

var **Nt** : Logic; Normality test

**[** End Parameter Definition **]**

***PROGRAM START***

Parameter Reading: **S,** $\boldsymbol{L}_{\boldsymbol{\infty}}$**,** $\boldsymbol{k}$ **and** $\boldsymbol{\Delta}_{\boldsymbol{i}}$, $i=1,2,3,4$

**[** Data Reading and Statistical Treatment **]**

# *Accessing the database*

**temperatureData** 🡨 dataReading("Database")

# *Normality Test on the data*

**Nt** 🡨 testNormality(**temperatureData,** **Shapiro-Wilk**)

**[** End Data Reading **]**

**[** Confidence Interval **]**

% This will be used in the definition of the fuzzy set for average temperature

**if** Nt **is true**

**CC** 🡨 “select 400 points from the interval [0.01,1]”

**for k=0 to len(CC)**

$\beta$ 🡨 **CC[k]**

**IC[k]** 🡨 “Confidence Interval to Confidence Level 1-$\beta$”

**End for**

**End if**

**[** End of Confidence Interval **]**

**[** Calculation of the Average Temperature Fuzzy Set $\tilde{T}$ **]**

function **tempFuzzySet** (IC,CC)

**for i = 1 to length(CI)**

$\boldsymbol{\alpha}$ **🡨 CC[i]** % alpha-levels

$\left[ \tilde{\boldsymbol{T}} \right]^{\boldsymbol{\alpha}}$ **🡨** **CI[i]** % Construction alpha-levels set

**End for**

**return (**$\tilde{\boldsymbol{T}}$**)**

**End** function

**[** End Calculation of the Average ... **]**

**[** Membership Function for Average Annual Temperature Values ​​under IPCC scenarios **]**

**for j = 1 to 4, do** # variation of scenarios

**for i = 1 to length(CI)**

$\boldsymbol{\alpha}$ **🡨 CC[i]** % alpha-levels

$\left[ {\tilde{\boldsymbol{\Delta}}}_{\boldsymbol{j}} \right]^{\boldsymbol{\alpha}}\boldsymbol{=}\boldsymbol{\Delta}_{\boldsymbol{j}}$ % alpha-levels to IPCC scenarios

**End for**

**End for**

**for i = 1 to 4, do** % Variation of scenarios

${\tilde{\boldsymbol{T}}}_{\boldsymbol{i}}$ **=** $\tilde{\boldsymbol{T}}$ $\boldsymbol{\bigoplus}$ ${\tilde{\boldsymbol{\Delta}}}_{\boldsymbol{i}}$ % Sum of fuzzy sets

**scenarioGroups 🡨** ${\tilde{\boldsymbol{T}}}_{\boldsymbol{i}}$ % Stores set informations

**End for**

**[** End Membership Function for Average ... **]**

**[** Membership function degree of the fuzzy subset $\tilde{T}_{i}$ **]**

% Returns the membership function degree of the temperature to the set $\tilde{T}_{i}$

function **degreeBelongsTemp** (tmptr, $\tilde{T}_{i}$)

**if (“**the **tmptr** exceeds the support of the Fuzzy Set ${\tilde{\boldsymbol{T}}}_{\boldsymbol{i}}$**”**)

**return 0**

**else**

**return** (“The degree to which the element **tmptr** belongs to the fuzzy set ${\tilde{\boldsymbol{T}}}_{\boldsymbol{i}}\boldsymbol{"}$)

**End** function

**[** End membership function degree **]**

**[** Inverse Function for mortality **]**

**# USED PARAMETERS**

**# valueM** : Real; Numerical value for mortality

**#** $\boldsymbol{invF}\boldsymbol{p}$ : Object containing the inverse function obtained with Pauly's equation

**# Returns the temperature value associated with the mortality value**

function **invMortality** ( valueM, K, $L_{\infty}$)

% Inverse function obtained with Pauly's equation

$\boldsymbol{invF}\boldsymbol{p}$ 🡨 Inverse FunctionPauly(K, $L_{\infty}$)

**tmptr** = $\boldsymbol{invF}\boldsymbol{p}(\mathrm{valueM})$

**return tmptr**

**End function**

**[** End Inverse Function for Mortality**]**

**[** Calculates Fuzzy Set for Mortality using Zadeh's Extension **]**

**# USED PARAMETERS**

**# list_M** : array; stores mortality values

**# list_Degree** : array; stores membership values ​​relative to mortality values

**# value_Mort** : Real; mortality value

**# value_Temp** : Real; temperature value

**# degree_Mort** : Real; mortality membership function degree

**for c = 1 to len(grupoCenarios), do**

**var list_M** : : Defines the array that stores mortality values

**var list_Degree** : : Defines the array that stores to mortality values

**for m in range(len(S))** % Space of mortality values

**value_Mort** 🡨 **S[m]**

**value_Temp** 🡨 invMortality(**val_Mort**)

**degree_Mort** 🡨 degreeBelongsTemp(**val_Temp**, **scenarioGroups[c]**)

**list_M** 🡨 Stores the value of mortality(value_Mort)

**list_Degree** 🡨 Stores the membership values(degree_Mort)

**End for**

**End for**

**[** End Calculates Fuzzy Set for Mortality **]**

***END OF PROGRAM***
